# Gradenigo's syndrome presenting as IX and X cranial nerve palsy without clinically apparent ear infection: A case report and review of literature

**DOI:** 10.1016/j.ensci.2022.100397

**Published:** 2022-03-17

**Authors:** Safia Bano, Ahmad Nawaz, Abyaz Asmar, Muhammad Aemaz Ur Rehman, Hareem Farooq, Hamid Ali

**Affiliations:** a Department of Neurology, Mayo Hospital, King Edward Medical University, Lahore 54000, Pakistan; b Department of Neurology, Medical University of South Carolina, United States

**Keywords:** Case report, Cranial nerve palsies, Gradinego's syndrome, otitis media, Otorhinolaryngologic diseases, Petrositis

## Abstract

Gradenigo's syndrome (GS) is a triad (otorrhea, abducens nerve palsy, and pain in the trigeminal nerve distribution) of clinical findings that are caused by contiguous spread of petrous apicitis to the nearby neurovascular structures. Petrous apicitis is usually secondary to otitis media but atypical etiologies and absence of the classical triad pose a diagnostic challenge for physicians. We report a rare case of GS in an afebrile 55-year-old male who presented with unilateral headache, dysphagia and hoarseness (IX and X cranial nerve involvement), and diplopia with lateral gaze palsy (VI nerve involvement) in the absence of trigeminal neuralgia or a history of otitis media. Magnetic Resonance Imaging (MRI) revealed hyperintense lesions in the right petrous apex indicating petrous apicitis, the hallmark of GS. Prompt initiation of broad-spectrum antibiotics led to a marked improvement in dysphagia and voice quality on the 4th post-admission day, and complete resolution of symptoms by the end of the fourth week. This shows that GS can present even in the absence of clinically apparent ear infection and cranial nerve palsies may not be limited to the V and VI nerve in all cases. Physicians should be aware of such atypical manifestations as prompt radiological assessment followed by early antibiotics can prevent life-threatening complications from developing.

## Highlights

• Gradenigo's syndrome is a triad of otorrhea, facial pain and VI nerve palsy.
• The classic triad is seen in less than half of the cases.
• Petrous apicitis is the hallmark radiologic finding.
• The history of ear infection may not always be evident.
• Presentation with IX and X nerve palsy can pose a diagnostic challenge.

## 1 Introduction

Gradenigo's syndrome is a constellation of clinical symptoms classically caused by the spread of otic infection into the apical part of the petrous temporal bone (petrous apicitis) [1]. Due to the proximity of neural and vascular structures, the inflammation can extend into Dorello's canal (contains abducens cranial nerve and inferior petrosal sinus) and Meckel's cave (contains ganglion of the trigeminal nerve). This results in a classic triad of symptoms (otorrhea/ear pain, abducens nerve palsy, and pain in the trigeminal nerve distribution) known as Gradenigo's syndrome. In 1904, Giuseppe Gradenigo first identified and described this classical triad, but various cases have emerged with atypical manifestations [1,2], suggesting that the description of Gradenigo's syndrome as a classic triad may need to be re-visited. Neuroimaging i.e., Computed tomography scan (CT scan) and Magnetic Resonance Imaging (MRI), is employed as part of the diagnostic workup in all suspected cases [1]. Even in cases with an atypical presentation, the involvement of petrous apex is almost always reported on imaging, suggesting that petrous apicitis forms the cornerstone of definitive diagnosis [3]. Conservative management with antibiotics is usually preferred while surgery is reserved for refractory cases [4]. In this report, we discuss a radiologically diagnosed case of Gradenigo's syndrome in a 55-year-old male, who presented with VI, IX, and X cranial nerve palsy in the absence of fever and otitis media, and was successfully treated with antibiotics and steroids.

## 2 Case report

### 2.1 Case description

A 55 years old Pakistani male was admitted to the Neurology Department of a tertiary care hospital with complaints of right-sided headache, difficulty swallowing (dysphagia), nasal regurgitation, and hoarseness of voice. The headache had been present for the last three months whereas the other complaints developed five days after the onset of the headache. The dysphagia was initially limited to liquids but progressed to solids within two days. He also complained of diplopia on the rightward gaze for the last 15 days. However, the patient did not report any history or active complaint of hearing loss, ear pain, or discharge. He was normotensive and nondiabetic with no history of smoking.

### 2.2 Physical examination

On general physical examination, the patient was afebrile with normal vital signs. Ophthalmologic examination revealed an inability to abduct the right eye and double vision with a rightward gaze. However, the remainder of the extraocular movements were normal. His visual acuity was 6/6 in both eyes with normal color perception and contrast sensitivity. Pupils were equally reactive to light bilaterally and dilated fundus examination was normal with no afferent pupillary defect. On examination of IX and X cranial nerves, the soft palate raised symmetrically but the uvula deviated to the left, and the gag reflex was impaired. Indirect laryngoscopy showed vocal cords in a mid-adducted position. On otoscopic examination, the tympanic membrane appeared normal and there were no signs of effusion or discharge, and audiometry was normal. The patient's facial nerve was intact and no facial asymmetry was noted. The remainder of the systemic examination was unremarkable.

### 2.3 Investigations

**Fig. 1:**
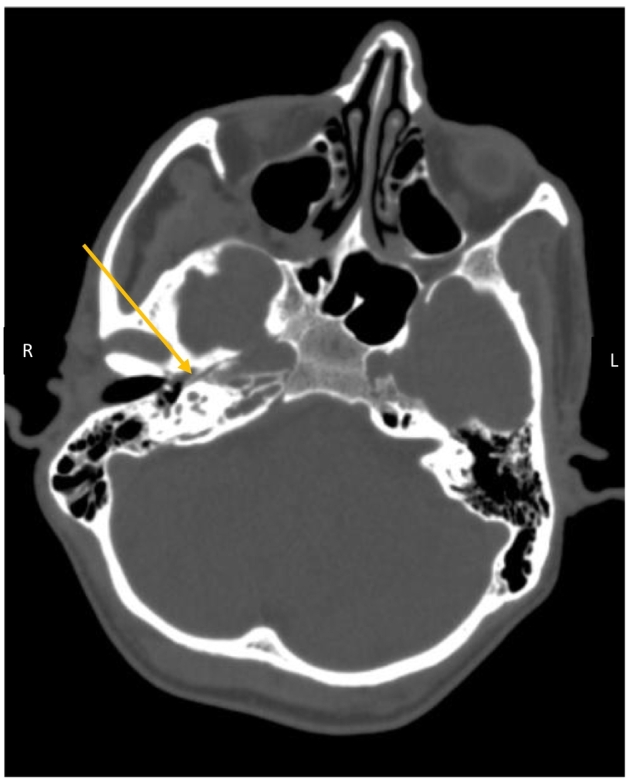
CT Brain (plain) shows suspicion of right sided petrous apicitis and otitis media.

**Fig. 2:**
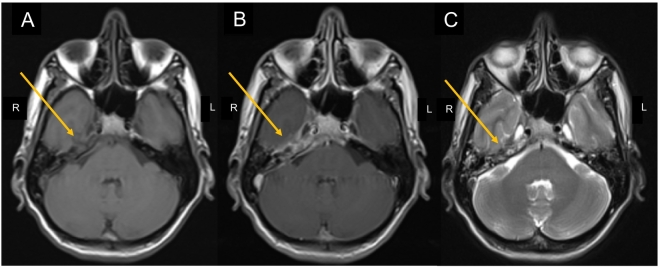
(A) MRI T1-Weighted image showing hypointense right petrous apex as compared to the left. (B) On enhanced axial T1-Weighted MRI image, prominent enhancement is seen on right petrous apex. There is also mild dural contrast enhancement in the temporal region without evidence of leptomeningeal involvement or parenchymal involvement. (C) MRI T2-Weighted image showing hyperintense right petrous apex in comparison with the contralateral petrous apex.

All routine baseline investigations were normal, except for Total Leukocyte Count (TLC) and C-reactive protein (CRP) which were raised. Initial imaging through a Computed Tomography (CT) scan revealed partial opacification of mastoid air cells bilaterally, with the right-sided opacification extending to the petrous apex. (Fig. 1) T2-weighted (T2W) MRI of the brain with contrast revealed hyperintensity of the right petrous apex with the corresponding low-intensity signal on the T1-Weighted (T1W) sequence. (Fig. 2) Post-contrast images showed prominent enhancement of the right apex along with mild dural contrast enhancement in the right temporal region without any evidence of leptomeningeal or parenchymal involvement. These radiological findings were suggestive of petrous apicitis. Nerve Conduction Studies (NCS) were also carried out to rule out the alternative diagnoses.

### 2.4 Differential diagnosis

Based on the patient's age, headache, and sixth nerve palsy, we initially suspected a space-occupying lesion (SOL) near the jugular foramen (hence involving the IX and X nerves as well). Motor neuron disease associated with bulbar palsy was next on the differential list. However, these were ruled out on the basis of lack of radiological evidence, normal NCS, and an incomplete clinical picture. Our patient's right petrous apex enhancement on T1W MRI imaging, coupled with his subacute presentation, cranial nerves palsies, and raised TLC and CRP (suggesting silent infection) led to the diagnosis of an atypical Gradenigo syndrome, i.e., without otitis media and orbital or facial pain (trigeminal involvement).

### 2.5 Treatment

On suspicion of an SOL, the patient was initially treated with intravenous dexamethasone 8 mg thrice a day for 10 days. Intravenous antibiotics were started on the fourth day of admission based on the radiological report (suggesting petrous apicitis) and included ceftriaxone 1 g twice a day and piperacillin/tazobactam 4.5 g thrice a day for 4 weeks.

### 2.6 Outcome and follow-up

**Fig. 3:**
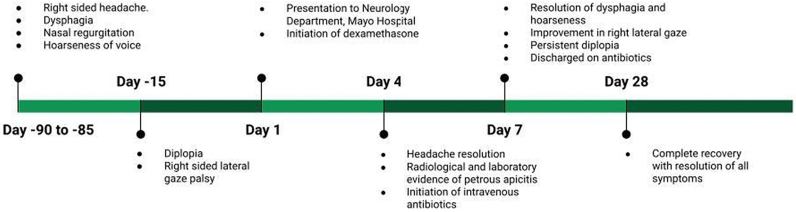
Timeline of events.

Significant clinical improvement was observed with the prescribed treatment during one week of hospital stay. Headache resolved by the fourth day, whereas restricted abduction of the right eye, dysphagia, and voice quality significantly improved by day 7 of hospital stay. Some degree of diplopia, however, persisted. The patient was discharged on ceftriaxone and piperacillin/tazobactam for a further three weeks, and he achieved a complete recovery 28 days post-admission. The complete timeline of events is shown in Fig. 3.

## 3 Discussion

Otitis media (OM) is found to be responsible for a variety of intracranial and extracranial complications [5]. Gradenigo's syndrome (GS) is one of the rare complications of OM's intratemporal spread that was first recognized by Gradenigo as a condition presenting with unilateral facial pain (V nerve involvement), lateral gaze paralysis (VI nerve involvement), and otorrhea [6]. Various atypical manifestations like otic meningitis, VII nerve involvement, and lack of otorrhea have also been reported in the literature, so the classic triad has largely fallen out of favor [3,7,8]. Given the anatomical contiguity of neural structures, and our limited understanding based largely on case reports and case series, a spectrum of atypical clinical presentations are likely to be reported in the future as well.

The usual cases of GS are due to the spread of middle ear infection into the temporal bone, eventually causing apical petrositis in patients whose apex is pneumatized [9]. Besides ear infection, other causes of GS that have been reported in the literature include neoplastic lesions (e.g., nasopharyngeal carcinoma, metastatic non-Hodgkin lymphoma), tuberculous apicitis, etc. [3,10] Regardless of the etiology, a common pathological theme in GS is inflammation and spread to nearby neural structures. In our case, the likely underlying pathology of petrous apicitis without a history of otitis media or fever might be explained by silent or asymptomatic OM. This is further supported by the fact that antibiotics given to the patient improved his symptoms drastically, and the CT scan of our patient also suggested a slight degree of otomastoiditis. Another possibility is an increased interval between otologic symptoms and cranial nerve complications that resulted in the patient's inability to recall or link these symptoms with his presenting complaint. Involvement of the IX and X cranial nerves which was found in our case in the form of dysphagia and hoarseness can likely be explained by the extension of inflammation into the skull base or jugular foramen [11].

The review of the available literature also shows that not even half of the reported cases present with the classical triad of symptoms, hence making it challenging to diagnose this disease [12]. In addition to the fifth and sixth cranial nerves, involvement of additional cranial nerves has also been observed [2]. However, presentation with IX and X nerve palsy without otorrhea and trigeminal nerve involvement is very unusual and serves as a diagnostic challenge for physicians. C·V and Hasan have previously described a case of GS with orbital pain and double-vision in the absence of ear symptoms [7]. Out of the eight patients included in a case series by Chole and Donald, only one patient had cranial nerves IX and X deficits [2] Recently, Parekh and Pacheco reported a case of GS and Vernet's syndrome (IX, X, and XI CN palsy) that developed as a result of the extension of candidal OM into the petrous apex and jugular foramen [11]. These, and other atypical cases [2,3,7,8,10,11,[13], [14], [15]] of Gradenigo's syndrome reported in the literature have been summarized in Table 1.

**Table 1:** Literature review of cases of Gradenigo's syndrome with atypical presentation.

Atypical cases of Gradenigo's syndrome					
Author, Year	Atypical presentation	Etiology	Imaging modality/ Investigation	Management	Outcome
Parekh, et al. [11]	6th and partial 3rd nerve palsy initially, followed by progressive dysphagia, dysarthria, right otalgia, and hearing loss	Candida mastoiditis	MRI of the brain and orbits	Medical (antibiotics and antifungals) and surgical (debridement and mastoid biopsy)	Death after several weeks
Sathe, et al. [10]	Left-sided hemicranial headache and reduced sensation on the left half of face, afebrile	Tuberculous petrositis	HRCT temporal bone, MRI Brain, MR venogram, and MRI orbit	Medical (prolonged antibiotics) followed by surgical (cortical mastoidectomy)	Full recovery
Bowman, et al. [14]	Facial pain, otalgia, diplopia, dysphagia, hypophonia	Complicated otitis media	Brain MRI	Medical (antibiotics)	Significant improvement but vocal cord palsy persisted
Sumana C V et al. [7]	Headache and unilateral periorbital pain, no ear discharge but a history of ear pain	Complicated AOM	Brain CT Scan and MRI	Medical (IV antibiotics) and surgical (cortical mastoidectomy)	Full recovery
Taklalsingh, et al. [8]	Meningitis, CSOM, 4th, and 6th nerve palsy, numbness in the 5th nerve distribution	Complicated CSOM	Brain MRI	Medical (IV antibiotics)	6th cranial nerve palsy resolved after 3 months, 4th and 5th nerve palsy remained unchanged, cerebullomedullary abscess resolved
Macasaet et al. [15],	Left-sided otorrhea, cheek and jaw pain, otalgia, hoarseness, dysphagia, and diplopia, left vocal cord, lateral gaze, and facial nerve paralysis	Complicated CSOM (cholesteatoma)	CT scan (cranial and temporal bone)	Medical (antibiotics) and Surgical (canal wall down mastoidectomy)	Postoperatively, the otalgia and jaw pain diminished while hoarseness and lateral gaze palsy remained.
Pedroso, et al. [3]	Abdominal pain, mild headache, left 5th, 6th, and 7th nerve palsies, no otorrhea or history of OM	Metastatic non-Hodgkin's lymphoma to the petrous apex	Brain MRI	Chemotherapy	Partial improvement, ongoing treatment
Jana, et al. [13]	Ear discharge, hearing loss, unilateral headache, and retro-orbital pain, dysphagia, nasal regurgitation, 5th, 6th, 8th, and 10th nerve involvement	Nasopharyngeal carcinoma	CT Scan of temporal bone and nasopharynx	Not mentioned	Not mentioned
Chole, et al. [2]	Right ear pain, headache, hoarseness, dysphagia, right shoulder weakness	Complicated AOM	CT Scan of the base of the skull	Medical (antibiotics) followed by surgical (myringotomy, simple mastoidectomy, removal of infected bone, and Penrose drain placement)	The infection resolved but no improvement in the 9th, 10th,11th cranial nerves

OM: Otitis media; AOM: Acute otitis media; CSOM: chronic suppurative otitis media; MRI: Magnetic Resonance Imaging; CT scan: Computed tomography scan; HRCT: High-resolution CT Scan; IV: intravenous.

Based on an afebrile presentation with no active or previous history of ear infection, and due to headache with sixth cranial nerve palsy (a false localizing sign indicative of raised ICP) in an elderly patient, our provisional diagnosis was a space-occupying lesion (SOL). The presence of dysphagia and hoarseness raised suspicion of bulbar palsy secondary to motor neuron disease (MND) but it was ruled out based on normal findings of NCS. CT scan ruled out SOL and showed some degree of opacification in the petrous apex region on the right side. CT scan is considered to be the first-line imaging test for petrous apicitis and it usually detects the presence of disease in the mastoid and petrous apex [16]. Further details and the etiology of the lesion are revealed by high-resolution MRI [17]. We investigated the patient through MRI that confirmed our suspicion of petrous apicitis causing an atypical GS.

The latest guidelines recommend using medical management (intravenous antibiotics) over the long-established surgical procedures like radical mastoidectomy and labyrinthectomy [18]. Ideally, antibiotics should be chosen according to culture sensitivity but for initial therapy and culture-negative patients, empiric treatment should be preferred. A culture test was not ordered in our case; however, the patient responded to empiric broad-spectrum therapy covering the most commonly implicated organisms (*Pseudomonas, Strep Pneumoniae, Strep Pyogenes, Staph Aureus*) [9]. Since we provisionally suspected a neoplasm (leading to raised ICP), steroids were started initially but antibiotics were started soon after the radiological confirmation of the diagnosis, and steroids were gradually tapered. The role of steroids in GS is controversial but it may reduce edema that relieves nerve compression and help in speedy recovery. Management of GS with a combination of steroids and antibiotics has also been reported previously [19].

There are certain limitations that the authors would like to acknowledge. Owing to the absence of fever and otorrhea, a culture test was not performed in our case and broad-spectrum antibiotics (including *Pseudomonas* coverage) were administered empirically. There has been no consensus on the duration of prescribed antibiotics so the patient was given the medication for 4 weeks and improvement was clinically observed. Although some cases of GS have previously used follow-up radiological assessment (MRI) to confirm recovery, we used improvement in clinical symptoms to confirm the resolution. The rare set of GS symptoms observed in our case led to a presentation that closely mimicked an alternative diagnosis (e.g., Malignancy, MND), hence the educational value of our case for practicing physicians is paramount. Early radiological assessment and timely treatment led to the control of spread and prevention of further complications.

## 4 Conclusion

We reported an extremely atypical and diagnostically challenging case of Gradenigo's syndrome (GS). The classic clinical triad of ear symptoms, lateral gaze paralysis, and sensory impairment of the trigeminal nerve has largely fallen out of favor and may not be seen in most cases. GS can present without current or previous history of ear infection and may present with IX and X nerve palsy without involving the trigeminal nerve. It is essential for physicians to recognize such rare symptoms, as early diagnosis and treatment can reduce the complications and mortality of this clinical entity.

## Declaration of Competing Interest

The authors declare that the research was conducted in the absence of any commercial or financial relationships that could be construed as a potential conflict of interest.
